# A World of Gorse: Persistence of *Ulex europaeus* in Managed Landscapes

**DOI:** 10.3390/plants8110523

**Published:** 2019-11-19

**Authors:** Nicholas Broadfield, Melinda T. McHenry

**Affiliations:** 1 Geography and Spatial Sciences, The University of Tasmania, Sandy Bay, Tasmania 7005, Australia; 2 Sun Pharmaceuticals Australia, 14 Henry Street, Latrobe, Tasmania 7307, Australia

**Keywords:** woody weeds, fire, grazing, disturbance, cost-benefit, mycoherbicides, phytophagous

## Abstract

Gorse (*Ulex europeus* L.) is a woody legume and invasive woody weed that has been introduced to temperate pastoral landscapes worldwide. Despite the apparent cosmopolitan distribution of gorse across much of the temperate agroecological landscapes of the world, research and practice pertaining to the management of gorse has been largely constrained to single-treatments, regions, or timeframes. Gorse eradication has been widely attempted, with limited success. Using the PRISMA (preferred reporting items for systematic reviews and meta-analysis) method and a quasi-metanalytical approach, we reviewed the seminal ~299 papers pertaining to gorse management. We identified (i) the ecological characteristics of the species that predispose gorse to behaving invasively, and (ii) the success of management actions (from a plant ecological life history perspective) in reducing weed vigour and impact. A broad ecological niche, high reproductive output, propagule persistence, and low vulnerability to pests allow for rapid landscape exploitation by gorse throughout much the world. Additionally, there are differences in flowering duration and season in the northern and southern hemisphere that make gorse particularly pernicious in the latter, as gorse flowers twice per year. The implications of these life history stages and resistance to environmental sieves after establishment are that activity and efficacy of control is more likely to be favourable in juvenile stages. Common approaches to gorse control, including herbicides, biological controls, and fire have not been ubiquitously successful, and may in fact target the very site resources—sward cover, soil stability, hydrological balance—that, when degraded, facilitate gorse invasion. Ongoing seedling regeneration presents difficulties if eradication is a goal, but facilitated competition may reduce costs via natural suppression. Mechanical methods of gorse removal, though highly successful, induce chronic soil erosion and land degradation and should hence be used sparingly.

## 1. Introduction

**Figure 1:**
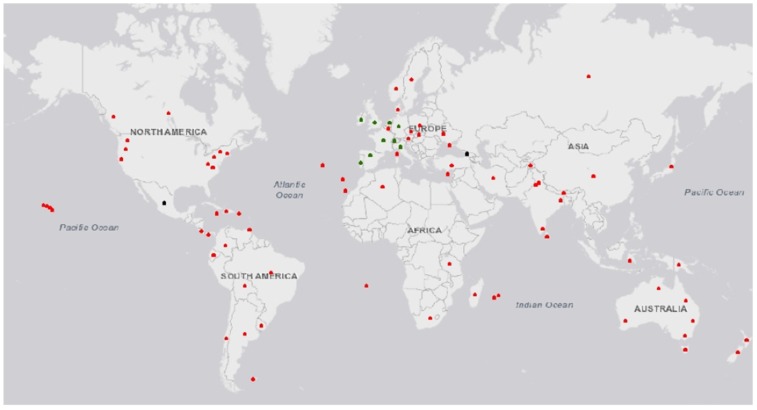
Reported global distribution of gorse. Green—natural distribution, red—introduced populations, black—unknown (presumed introduced). Created in ArcMap 10.5 from national and international databases (after [6]).

Common gorse (*Ulex europaeus* L.) is a thorny evergreen shrub that forms invasive thickets in pasture outside of its center of origin in Western Europe and Northern Africa [1]. First introduced for its potential as an ornamental, for fencing in agricultural landscapes, and for fodder, gorse is listed by the International Union for Conservation of Nature as one of the top 100 invasive plants on earth [2,3]. Due in part to accidental introductions due to soil translocation [4,5], gorse is now distributed from equatorial to temperate regions, but more successful in regions with limited exposure to seasonally extreme temperatures and annual rainfall between 500 and 1500 mm (Figure 1).

Biological, chemical, and mechanical interventions have been diverse and numerous, with none achieving ubiquity in controlling the negative effects of gorse, nor its large population size. Additionally, treatments for gorse often occur in singularity, or target-specific life history stages, without opportunity to examine longer-term outcomes, nor contextualise these within environmental or ecological constraints. 

Thus, in the present paper, we reviewed the outcomes of management actions used to control gorse, throughout time and across the world, with particular emphasis on the interactions between species ecology, treatment efficacy, and treatment impacts upon environmental stability. We used peer-reviewed literature to first synthesise the life history and ecology of gorse (Objective One), before reviewing management actions and their quantified successes and failures (Objective Two). We conclude this article by suggesting targeted management of gorse that reflects the ecology of the species and the environments that it is most successful in invading (Objective Three).

## 2. Findings

### 2.1. Life History and Ecological Success

**Figure 2:**
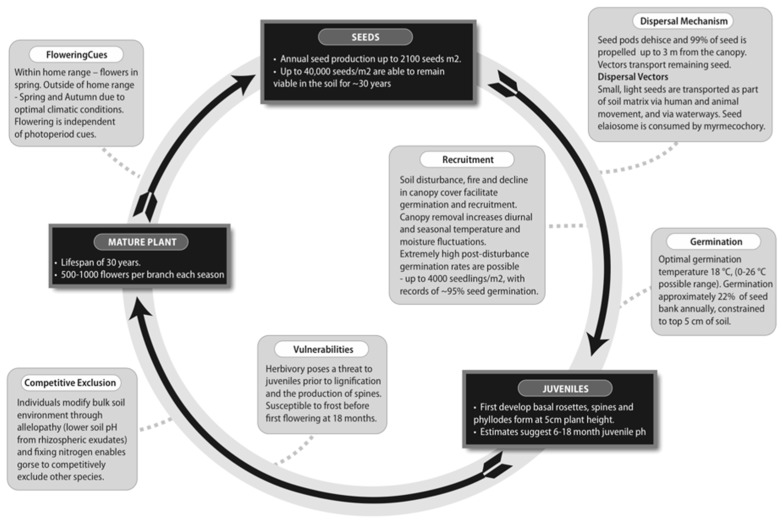
Life history traits of common gorse, including factors influencing the success of the species in a range of global environments.

The life history traits that facilitate the competitive success of gorse are persistent soil seedbanks and copious seed output, few constraints on recruitment, the ability to modify the surrounding environment to favour spread, and a short gap between juvenile phase and fecundity (Figure 2). 

Gorse colonises bare ground and more intact vegetation, such as grassy woodland. Approximately 6000–18,000 fertile seeds are produced annually [7,8,9] from mature individuals that develop approximately 1000 flowers per branch. Gorse flowers cannot self-pollinate, ensuring outcrossing and genetic variation [10]. Gorse flowers in both winter and spring, avoiding seed feeders during the spring that can prevent up to 90% of seed production [11,12]. In the absence of seed feeding predators, seed set is almost 100%. Seed pod maturation takes 8 weeks. Mature seeds are dispersed throughout the landscape initially by the explosive dehiscing of pods (each containing up to 12 seeds), scattering them up to five meters from the source plant [13].

Soil seedbank viability varies between regions and soil depths, with estimates of 20 years of seed longevity in the top five cm of soil, and significantly longer lifespans for seeds deeper in the profile due to reduced temperature fluctuations [14]. Due to seedbank lifespan and an annual seed fall from mature stands of 500–2000 seeds per m^2^, soil seedbank size can exceed 10,000 seeds per m^2^ [15,16,17]. 

Gorse seeds germinate under a wide temperature range (0 to 26 °C in vitro—commensurate with observed live field emergence data [18]). Yet, mature, well-dried seeds sometimes have poor germination rates (~22% per annum), especially at lower soil depths [17]. Lower germination rates are thought to be the due to the hard, water-resistant seed coating that creates a physical barrier inhibiting seed development [17,18,19]. Disturbances and scarification can stimulate dense germination of ~2000 seedlings per m^2^ [19] and >95% success [17].

Gorse has a short juvenile period of 4–6 months from germination. Small leaves continue to develop until the plant is roughly 5 cm tall, ceasing when the first spines are produced [20] reducing vulnerability to herbivores. Fixation and uptake of soil nitrogen in nitrogen-deficient environments also facilitates the competitive success of gorse (as a leguminous shrub) over many other plant species [21,22,23] and modifies rhizosphere pH. 

Gorse matures and flowers ~18 months after germination. Temporal variation in flowering phenology occurs in many places, with year-round flowering occurring independently of photoperiod cues [9]. In general, however, flowering takes 2–3 months, with flowers developing in synchronised flushes to increase the prospect of cross-fertilisation [24]. Flowers are unable to self-pollinate. Insects probe the keel base for nectar, depositing pollen on the insect’s ventral surface so as to maximise opportunities for out-crossing [9]. Mature gorse plants live for up to 30 years, and flower at least once annually from their second year of life.

The spread of gorse into a range of environments has been facilitated by a range of dispersal vectors. A range of agroecological disturbance regimes also create conditions conducive to further invasions. Success may be enhanced by liberation from coevolved predators [24,25]. 

Modification of vegetation cover, soil disruption, and fire increase seed germination. The removal of vegetation may stimulate seed germination through the increased variability in diurnal and seasonal soil temperatures and moisture content [26]. The disruption of the soil caused by animal passage, soil engineers, and human activity causes mechanical scarification of the seed coat, enabling seeds to germinate freely [16]. Fire also results in increased germination [19]. Even short exposure to temperatures of up to 110 degrees Celsius for 5 min significantly increase germination [27]. As soil is an effective insulator (dissipating the heat and producing conducive temperatures for seed germination just below the surface), the hottest of fires are unlikely to destroy the seedbank [19,20,21,22,23,24,25,26].

In addition to dehiscing of seed pods, seeds are dispersed naturally by ants which are attracted to the protein-rich elaiosome [20]. Animals, livestock, and humans act as soil seedbank transport vectors [28]. The most significant dispersal vector, however, is water. Peak flows rapidly spread gorse seed downstream [29].

### 2.2. Management of Gorse—An Ecological Perspective

**Figure 3:**
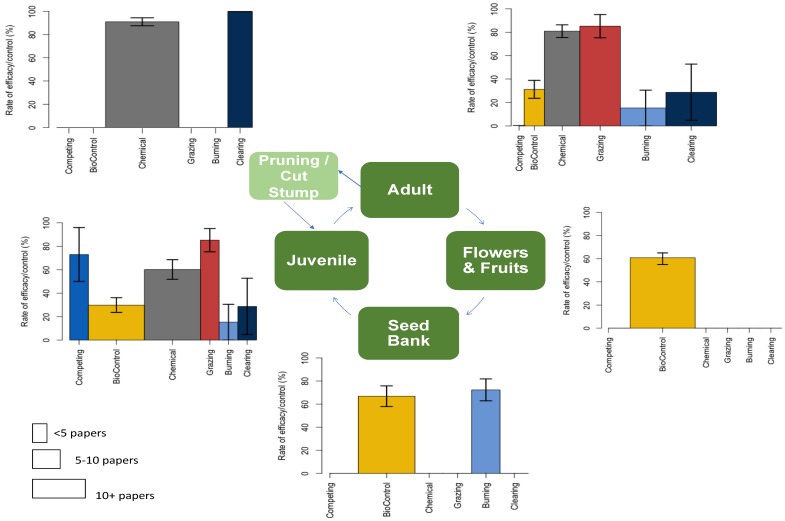
Rate of reported success (±S.E.) and paper number (*x*-width) of various management actions (royal blue = competition; yellow = biological control; grey = chemical controls; red =grazing; light blue = burning; indigo = clearing) targeted at different lifecycle stages of common gorse: competition; biological control; chemical herbicides; grazing; burning; land clearing.

Management actions have primarily focused on the mechanical removal of mature plants and/or juveniles, limiting seed production, impeding plant regeneration, causing foliar damage and reducing plant longevity, and the suppression of seed germination and seed banks in the landscape (Figure 3). Chemical herbicides (including those that were painted on cut stumps) and biological controls were the most popular treatments for which trial data could be found in published literature. The most diverse treatment options were evaluated in juvenile and adult stages, covering a broad spectrum of options from competition to fire and land clearing. Despite the relatively exhaustive literature search that underpinned this review, we were surprised to find that many seemingly ‘common’ management strategies for gorse were underpinned by very few published items, and that some treatments were demonstrated—repeatedly—to have had limited success, but continued to be trialed (e.g., biocontrols). Henceforth, we review the relative success and failure of these treatments.

#### 2.2.1. Competition

Interspecific competition appears to suppress gorse recruitment, at least in the early juvenile stage, and can be manipulated in production environments. Gorse suppression by competition was first described by [30], with gorse in its native range being unable to completely form closed canopies when in competition with bracken fern (*Pteridium esculentum*). Over-sowing recently cleared landscapes has also been suggested to reduce germination and success of gorse seedlings. Growth suppression has been demonstrated when accompanied by ryegrass in vitro [31] and when competing with white clover [32]. A 96% reduction in seedling dry weight (without a commensurate decrease in seedling survival) was also observed in Tasmanian (Australia) field conditions [31]. The measured effects of competition were reported in only a small number of papers in our search (*n* = 5), although many subsequent articles refer to competition as a possible strategy in the management of gorse (e.g., [3,33,34]). 

#### 2.2.2. Biocontrol

Two categories of agents have been successfully introduced into the non-native range for gorse—seed feeders and foliage feeders. These agents have had a varied effect, causing damage to the reproduction and longevity of plants (Appendix A Table A1; Table A2), but are ultimately the primary target of the fruiting and seed production (and subsequent germination) stages of the gorse lifecycle. 

Seed-feeding invertebrates are unable to cause the suppression of weed populations, but nonetheless impede the reproductive output of individual plants [35]. Of the three seed-feeding agents released, only the pod moth, *Cydia succedana*, and seed weevil, *Exapion ulicis*, were successful. The pod moth is bivoltine with active larvae in autumn and spring that consumes seeds [36]. Damage to New Zealand seed production varied greatly, with 3%–60% mortality [9,36]. Seed weevils reportedly damage 70% of total pods, reducing 0%–92% of seed production across British evaluation sites [37]. A following study in its native range also found variably high rates of seed predation, with 35%–70% of seeds damaged [38]. Following the discovery of the capacity of the gorse seed weevil to limit seed production, it was released and monitored in many countries, including Australia, the USA, Chile, and NZ [39]. However, as the seed weevil is univoltine, it is only active during the spring flower production, thus being most effective during the sole flowering period in its native range [40]. When combined, *C. succedana* and *E. ulicis* destroyed virtually all spring seed and approximately 10% of autumn seed production, culminating in an annual reduction in seed fall of up to 90% [41,42]. 

Though these successes have indeed been moderate, the level of control of seeding obtained from these biocontrols appears insufficient to impede the persistence of gorse relative to the costs of development, breeding, release, and maintenance of the agents.

Foliage-feeders (Phytophagous species) are invertebrate species that suppress growth (and therefore vigour and often invasive potential) via the consumption of the photosynthetic plant organs. These species generally cause localised damage to the plant, especially in the juvenile stage, impeding vigour and reducing plant longevity [43]. However, it should be noted that 14 biological controls have been released since 1940 [44]. Of these, the spider mite (*Tetranychus lintearius*), soft shot moth (*Agonopterix umbellana*), gorse thrips (*Sericothrips staphylinus*), and the soft shoot moth (*Pempelia genistella*) have been established in at least one invasive gorse population [45]. Subsequent [46] research has suggested that host–pest interactions are highly complex and site-specific.

Each of the four phytophagous biocontrols has a different mechanism of action and has been adopted in slightly different regions. Introduced into NZ, Australia, the USA, Chile, and St Helena for the biological control of gorse, *T. lintearius* causes severe damage [42] via the production of large colonial, web-like entities that enable the population to use their stylets to suck out the entire contents of mesophyll cells causing cell death [5,47]. Once established in a stand, the spider mite has shown potential to kill individual shoots and reduce growth rates, with dry matter production reduced by 36%–44% and reduction in shoot elongation and flower production by 37%–82% [48]. The moth, *A. umbellana*, was introduced into NZ, Chile, the USA, and Australia [44] for the purpose of laying larvae that parasitise new spring shoots, thus reducing plant vigour [49]. The moth has sub-lethal effects, seasonally attacking and causing plant stress, decreasing maximum plant age [44].

Gorse thrips introduced into NZ, the USA, and Australia feed on both mature and juvenile vegetation [50]. Finally, the pod moth *P. genistella* is a univoltine species introduced to NZ and the USA, producing larvae that feed on mature gorse foliage in autumn. Larvae form small colonies and produce webs up to 30 cm in diameter, before overwintering and completing their development on the new, more nutritious spring foliage [9].

Despite some success in small trials, the results of each biocontrol have been equivocal. Although having a profound impact on individual plant health, in all populations (excluding those in Chile), native predatory mites suppressed spider mite populations, reducing the biological control of gorse [50,51]. No introduced population of *A. umbellana* has successfully caused any substantial damage, with the Chilean [52] and New Zealand [53] populations not establishing, a Tasmanian (Australia) trial population only being recorded in low population density [54], and the others being established yet undocumented since 1990 [41].

Although thrips have caused high seedling mortality with correspondingly high population rates in vitro [5], poor establishment in the field potentially due to natural enemies reduced the prospective impact of this biocontrol [34,55]. Finally, the impact of the hard shoot moth on plant populations is not considered significant, especially as introductions resulted in poor establishment and remaining progeny residing only in small populations [5,41]. Therefore, biocontrols may consume more effort in their development and maintenance than the target host.

#### 2.2.3. Herbicides

Herbicide application to juvenile foliage facilitates effective uptake [56]. Foliar herbicides seldom control mature stands, unless added surfactants can penetrate the thick, waxy cuticles of mature foliage [56,57]. An alternative to foliar applications in mature plants is the chemical treatment of cut stumps, first requiring the removal of above-ground vegetation. Herbicides applied directly to cut stumps are extremely effective, with the use of synthetic auxins being of highest proficiency (Table 1). Soil-applied, pre-emergent herbicides (although unable to penetrate the soil surface) have shown to be extremely effective in lab experiments, with synthetic auxins causing highest seed mortality [17]. Nonetheless, impacts and effects of herbicides are variable and their use is restricted in many temperate gazing landscapes.

Our research also revealed the increasing potential of mycoherbicides (herbicides of fungal origin). Two mycoherbicides *Fusarium tumidum* and *Chondrostereum purpureum*, have been evaluated and suggested to be possible non-synthetic chemical controls for plant regeneration (Appendix A Table A3). The development of *F. tumidum* as a mycoherbicide for the control of gorse first originated from the isolation of the pathogen on gorse in NZ [42]. The authors of [35] found leaves and flowers to be more susceptible to the pathogen than stems, spines, and pods, and the authors of [63] found *F. tumidium* to be able to reduce dry weights of shoots and roots of the adult plant.

Further studies examining soil and juvenile application found that such applications caused significant reductions in seedling emergence and 95% mortality in juvenile plants [64,65]. The pathogen *C. purpureum* causes ‘silver leaf’ disease and has been suspected to be the cause of localised declines in woody weeds [66,67], albeit that its specific mode of action as a mycoherbicide is unknown. The use of *C. purpureum* on cut stumps showed some degree of effectiveness in reducing the regrowth and survival of gorse plants, with the highest effectiveness seen in late summer and early spring application [5,68].

Mycoherbicides have only limited scope to cause significant mortality, and may in fact be deleterious to other populations of woody plants if introduced into non-target regions. Hill et al. [5] suggested that *F. tumidium* has only some effect on the control of gorse regrowth, having limited capacity as a cut-stump, foliar-applied remedy to gorse invasions, but that it could possibly be utilised in pre-emergent and juvenile stands in combination. *C. purpureum* is equally unlikely to be effective in managing plant invasions over large areas but may have potential as a mycoherbicide applied to regenerating gorse after mechanical removal [69].

#### 2.2.4. Grazing

Gorse has long been utilised for its apparent grazing potential, with livestock being able to selectively consume the plant when other fodder is in low supply. Notwithstanding the impacts of grazing intensity, common herbivores have different levels of efficacy when used as mechanisms of gorse control.

Cattle are least suited to the control of gorse, abandoning the fodder source once thorns have formed [70]. Fodder preference studies conducted on sheep and goats indicate a slight preference for the species by goats (65% versus 71% incidence of grazing, respectively) [71]. As gorse is a highly lignified woody weed, grazing by goats has been suggested to be an effective control method [72]. However, goats are of low production value, and thus approaches that combine ovines either in rotation or in mixed flocks can provide similar and effective reduction in gorse in the landscape with greater economic return [70]. Grazing can be enhanced through physical reduction of mature stands, allowing livestock access and the opportunity to graze young shoots.

#### 2.2.5. Burning

Fire as a management tool has been met with limited success [42]. Whilst sustained hot burns effectively remove above-ground cover, plants exposed to insufficient heat are able to rapidly re-sprout from roots and exposed stems [73,74,75,76,77]. Furthermore, the eradication of aboveground vegetation stimulates the germination of seeds stored in the soil seed bank [16,26].

Rolston and Talbot [19] found that seed banks were considerably reduced by hot burns, recording a seed bank decline of 62% in the top 10 cm of the soil profile. Yet, seeds buried below 2–5 cm were not killed [19], allowing later regeneration [78].

#### 2.2.6. Land Clearing

Land clearing, although effective in the interim, removes only the surface vegetation. This reduces canopy cover, facilitating rapid regeneration of the seedbank [8]. This rapid germination can be attributed to the increase in diurnal temperature variation, increased water penetration into surface soil (due to removal of canopy interception of water by shrub cover), and scarification the seeds, all allowing for the seed coats to degrade and germination to occur [17]. For effective mortality in adult gorse, plants must be cut off at least 5 cm below ground level, hindering rapid re-sprouting [61].

## 3. Implications for Practice

### 3.1. Linking Life History with Management Strategy

From our readings, we surmised the following:

1. Inhibition of gorse seed production, so as to avoid copious yearly (and in some places, biannual) seedbank additions, is desirable, but not practical through mechanisms either targeting fruits or the seed bank itself. Greater success could be afforded in environments where care is taken to prevent juveniles from recruiting into the adult population.
2. Biological controls and chemical herbicides are the most commonly trialed management actions, yet both have site-specific constraints. Additionally, biological controls were rarely effective, or required ongoing population maintenance.
3. Mechanical removal techniques such as land clearing and cut stump painting have immediate and demonstrable success, but cause secondary issues in soil disturbance and interference with nutrient cycles. These disturbances and perturbations are the same reasons as to why fire and grazing are largely unsuccessful in the medium- to long-term in controlling gorse, and why “managing for competition” might in fact be one of the best and most cost-effective long-term controls.

#### 3.1.1. Control of Seeds and Seedbanks

Like other woody weeds, gorse invasions are mediated by three processes, landscape invasibility, the inherent characteristics that enable the species to invade, and the propagule pressure on the landscape [79]. Temperate and modified landscapes (usually though agriculture and land clearing) provide a suitable environment for gorse invasion. Perhaps the most pervasive aspect of gorse invasiveness is via its copious seed production, and the high viability and temporal longevity of the soil seedbank maintains sustained propagule pressure within the landscape.

However, each of the strategies devised to reduce or target seed production or vigour comes with an equally compelling argument against it as a solution. Seed-feeding biological controls, especially *Exapion ulicis* [37,44,80] did indeed have moderate rates of success in reducing seed production and viability within the seed pod. However, these treatments were not effective in a second season without being combined with other, less effective treatments (such as mycoherbicides) in areas with two flowering periods because of the seed weevils univoltine feeding pattern. Weevil populations also required for ongoing maintenance.

Fire, the other evaluated treatment for the soil seedbank, relies on intense and frequent treatments to cause a significant reduction in *surface* seeds (0–10 cm), but (i) does little to damage seeds deeper in the soil profile, and, (ii) causes soil degradation and nutrient loss when used at sufficient intensity to cause surface destruction. Therefore, the use of fire at sufficient intensity to cause significant seedbank reduction also simply creates a bare and degraded landscape suitable for gorse reestablishment.

Thus, techniques to manage seeds and the seedbank may need to be labor intensive, or take many years of combined treatment protocols to reduce an initial seedbank accumulation. Seed production might need to be halted with an initial removal of juveniles and adult seeders using highly successful cut stump techniques (which do not always cause significant soil degradation), and progressively killing emergent seedlings on an annual basis, using minimally-invasive techniques such as herbicides (where possible), and occasional low-intensity fires to cause mortality to juvenile individuals and prevent recruitment into adulthood.

#### 3.1.2. Biological and Chemical Treatment

Biological controls using insects require population maintenance and absence of natural predators. Consequently, many of these have had only moderate success in affecting gorse vigour, and even less success as single-treatment reductions to seed populations and germination. Chemical applications and mycoherbicides were most effective on mechanically-removed vegetation that exposed a cut-stump, whilst soil-applied treatments resulted in poor germination suppression. Foliar herbicides were sometimes quite effective, but there are numerous restrictions on their use, including glyphosate in the EU and 2,4,5-T in North America [59]. Additionally, herbicides may affect non-target species [9], which is problematic when trying to maintain groundcover to suppress new growth of seedlings. These restrictions, alongside localised specificity testing of biological control agents [66,78], suggest that chemical and biological treatments may be highly site specific, or used as part of a broader suite of options in a longer term eradication strategy.

#### 3.1.3. Mechanical Treatments

Mechanical treatments are both the most successful options in causing instant mortality to aerial populations, and also the most likely to maintain conditions suitable for gorse re-establishment when used without care. The mechanical removal or destruction of gorse can induce chronic soil erosion, compaction, and structural decline, which decreases prospects for sustainable land rehabilitation.

Not all mechanical treatments involve land clearing, however, and some minimally invasive methods might include cut-stump and aerial mulching of gorse foliage. Methods that reduce soil impacts and seed bank disturbance (albeit that all gorse thicket removal will result in increased light and water supply to the upper soil seedbank) are both highly impactful and highly expensive.

### 3.2. Managing Gorse: Balance between Level of Disturbance and Level of Effort

The control of common gorse requires active management. This management is time-consuming and expensive, and thus must be targeted at life cycle stages that cause the most vulnerability in plant populations. As we have learned from previous sections, the most obvious life-history stages that define the success of gorse—seed production and seed storage—are currently those where we have the least amount of broad spectrum, successful options for mortality and control.

Before presenting a generalised strategy for gorse management, it is worthwhile noting additional ecological considerations and trajectories for the biotic potential of the species, and its future potential invasiveness in managed environments. Though it should be obvious that farm hygiene practices should be continuously observed to prevent repeat infestations [81], guidelines are in place in many countries but are not always enforced [15]. Additionally, climate change is predicted to increase disturbance events, and the likelihood of intense fire and flood events [28], each of which are postulated to be advantageous to gorse as an *r*-selected species [15].

Through our research, it is evident that gorse conforms with generalised weed invasion theory, where invasions can be predicted on the basis of the resource availability within the landscape, rather than specific landscape predictors. Thus, we must ascertain resource variables that best predict gorse distributions, enabling gorse to be effectively eradicated before establishment, and therefore decrease the probability of gorse either transforming landscapes or responding prolifically to deleterious landscape change. As a consequence of this research, it is apparent that the resource issues associated with gorse invasions are also those that result in negligible or negative effects of attempted treatments—soil exposure and declines in soil structure (such as after light fire, heavy grazing, and thicket clearing), changes to resource availability (modification of groundcover after fire or grazing, increased temperature, light and water penetration caused by land-clearing, overgrazing, or heavy fire), and removal of competitors (land clearing for agriculture, removal of competing vegetation by inappropriate fire, herbicide use or grazing).

**Figure 4:**
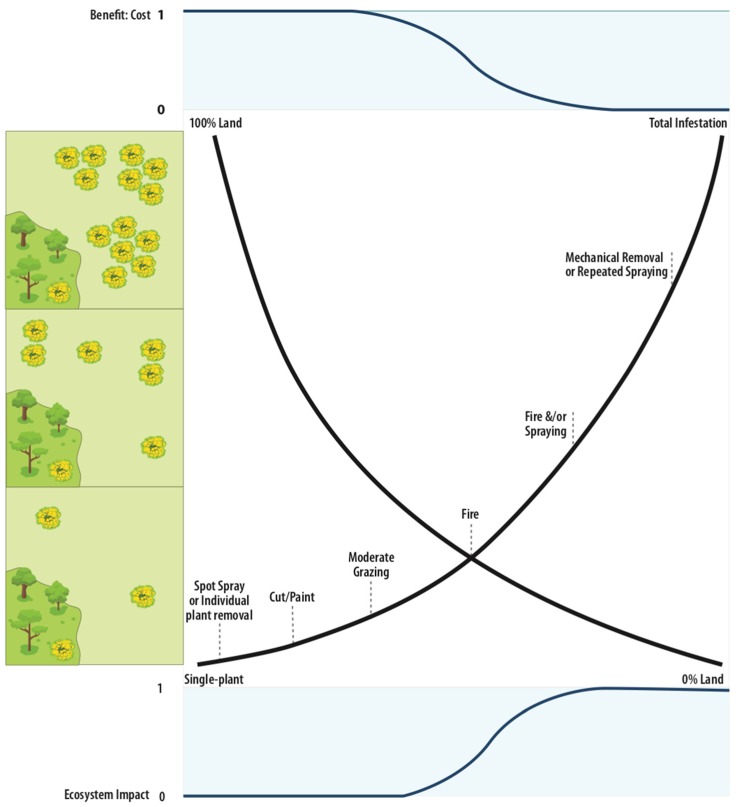
Management strategies for the control of gorse within landscapes, depicting each management strategy in relation to the density of gorse covering the landscape (bottom left to top right) and the proportion of the overall land area occupied (bottom right to top left). Increasing plant density is generally synonymous with more invasive management techniques, ranging from spot spraying of individual plants to mechanical removal of large infestations. With the use of more invasive techniques, the negative impact on ecosystem services increases rapidly (“ecosystem impact”, bottom of figure), whilst the associated benefit-cost ratio (top of figure) declines.

Therefore, in outlining a strategy for the management of gorse that incorporates economic and ecosystem vulnerabilities (Figure 4), we have classified treatments that have the propensity to affect ecosystem function as activities to be used either sparingly to clear thicket populations (that are dense and long-established), or when other, more expensive options are simply unavailable. By considering land clearing, grazing, and fire to be once-off measures, we have incorporated ecosystem vulnerabilities in a strategy that addresses current barriers to successful management. These barriers include the time and financial outlay of clearing gorse thickets [8] (whereby the cost of the initial removal of gorse in localised regions could exceed local land value [38]) and also the strategies that might need to be considered at various levels of gorse invasion (whereby large populations might only respond to more invasive techniques in the first instance).

In order to cause minimal disturbance, and when gorse population numbers are low, spot removal and competitive approaches should be considered. Despite initial reluctance of some land managers to spot-treat isolated individual or low-density infestations, considerable environmental and economic benefits are derived from investment in spot-spraying, individual plant removal, and grazing management. Even at higher population levels, it is evident that maintaining groundcover (even facilitated sward cover) is beneficial in suppressing emerging seedlings. All spot-removal and facilitated (or rehabilitated) competition approaches are only effective within the juvenile (or for isolated shrubs, non-fecund adult) stage, however, and should only be practiced as part of second or follow-up treatment if it was first necessary to remove a large and established population.

Once sufficient plants have been established, and recruitment has occurred, costs and propensity for landscape degradation increases proportionally and linearly. Fire and mechanical removal are more cost-effective strategies at the point of infestation/inflection, however, additional challenges are encountered in hilly terrains [82], riparian zones [15], and forested landscapes. Furthermore, mechanical removal of gorse in forested and grassy landscapes can be indiscriminate in vegetation removal. It is for this reason that herbicide usage is also not recommended as a primary management strategy once floral initiation has commenced.

At higher initial population densities, more intensely degrading measures might be required as part of a once-off vegetation-removal strategy. We consider fire to represent the ‘middle ground’ between cost, environmental impact, and level of infestation. When used appropriately, fire is low cost, has moderate and transient environmental impacts (at low frequency and intensity), but has limited effect beyond damaging or causing mortality to standing vegetation. For this reason, infestations of gorse should be at least moderately high before fire is considered a strategy to either remove mature plants, or to germinate the seedbank en masse so that it can be subsequently treated with foliar herbicides.

As it is in the interests of preventing future infestations of gorse in highly-suitable (i.e., disturbed, modified, or degraded) environments, it is recommended that large and dense populations be subject to mechanical removal only as a last resort. The subsequent germination of the seedbank should be managed in the first instance with shelter from grazing, fire, and interference (to non-target adjacent species) from herbicides, so as to facilitate competitive suppression. Spraying might only become an option if suppression is ineffective and plants are leaving the juvenile stage.

The salient point from this section is that management options that at first appear quite labor-intensive (cut stump) or insufficiently intensive for preventing recruitment (e.g., competition and suppression in seedlings) may ultimately result in lower longer-term costs and follow-up due to the maintenance of the ecological environment that results from lower levels of disturbance.

## 4. Materials and Methods

### 4.1. Methodological Approach

We conducted a systematic review of peer-reviewed literature on gorse ecology and management actions (with particular reference to peer-reviewed management trial literature) using the PRISMA (preferred reporting items for systematic reviews and meta-analysis) approach [83].

We interrogated Scopus, Web of Science, and Google Scholar databases, using (initially) only the search term ‘gorse *Ulex europaeus*’. Initially, 7000+ articles were identified, then we refined our search by explicitly removing irrelevant or duplicate articles and those unavailable in English. Most of the irrelevant articles were about the use of gorse in pharmaceutical products for human health, and a smaller subset were genomic studies.

Upon removal of irrelevant and duplicate articles, 179 articles that pertained specifically to gorse ecology and management remained. An additional 120 sources were later identified from citations within the initial 179 references found on Scholar, Web of Science, and Google Scholar databases, expanding the literature sample to a total of 299 published articles, peer-reviewed technical reports, and book chapters. It was our intent to be as exhaustive as possible in our literature search, and hence our data collection was constrained only by peer review and a lack (where relevant) of capacity to translate older articles into English. Therefore, our final sample contains articles and works published as early as 1896, and as late as January 2019.

### 4.2. Data Analysis

In total, 299 articles were analysed within NVivo Pro 11 [84]. NVivo Pro is a qualitative research analytical tool, which can be used, among other things, by the user to cluster text selections under distinct themes and ideas, to show relationships between themes and ideas, and to quantify literature sample proportions and dataset sizes.

As part of Objective One, we used life history stages (seeds, juveniles, and mature plants) as interpretive checkpoints for the successful management of gorse. We interrogated our NVivo dataset, searching for articles containing the words (and stemmed words related to each search parameter including plurals or pronouns) ‘seeds’, ‘seedlings’, ‘germination’, ‘flowers’, ‘juvenile’, ‘recruitment’, ‘maturity’, ‘senescence’, and ‘competition’. Literature sections, paragraphs, and documents containing these search terms were coded as individual “nodes”. We then conducted our reading from the literature contained within each node.

Objective Two saw us interested in the type, success, and/or failure of management activities to control gorse. Again, using an NVivo keyword search, we located complete and stemmed words from the terms: ‘biocontrol’, ‘clearing’, ‘cutting’, ‘removal’, ‘mechanical’, ‘fire’, ‘burning’, ‘chemical’, ‘herbicide’, ‘survival’, and ‘mortality’, which we coded as nodes. We then used matrix coding to cross tabulate and quantify the type of management actions against each identified life history stage, using the ‘life history’ and ‘management’ nodes.

So as to satisfy Objective Two, we conducted a quasi-meta-analysis, interrogating our matrix-coded, cross-tabulated dataset of compiled literature in the ‘life history’ and ‘management’ nodes for specific, quantified records of plant or plant part mortality, damage, or reduction in seed germination. We categorised our findings into life history stages (seeds, seedlings/juveniles, mature plants, flowers, and fruits) using summary statistics such as counts (of treatment types and attempts at various life history stages) and means (of percentage ‘success’ (±Standard Error – ‘S.E.’) in reduction of mortality or reproductive vigour) to quantify relative efficacy of herbicide, fire, mechanical, and biological control treatments across these stages.

## 5. Future Perspectives

In targeting weed invasions, it is important to not only target the symptoms, but the root cause of the invasion. Though gorse has a high biotic potential, and high propensity for landscape invasion due to its ecological strategies and successful life history, its persistence in the landscape can be minimised with careful management, as well as maintenance of ecosystem balance. Gorse, like many other woody weeds of production environments, is facilitated by modified resource use, and available space for germination and establishment. Maintaining swards, soil structural integrity, and paying attention to the frequency of disturbance regimes and intensities of fire, land clearing, and grazing is just as important as choosing a strategy for active management of gorse from spot removal to cut/paint to intermittent herbicide use, occasional grazing, or cool fire.

The future use of biological controls should also be reviewed. Biological controls have cost-considerable efforts in time, research, and financial inputs with very little reward on a large spatial or temporal scale. Many land managers simply do not have the time or resources to dedicate to biological controls if the seedbank will still fill with seeds, no matter how few, in the following season or year.

Both conservation professionals and farm managers should consider the maintenance of ground cover and a reduction in planned disturbances as the most important future strategies for control of gorse once active management strategies have taken affect in adjacent areas. Cover can be maintained naturally by excluding grazing animals in early seedling re-establishment, or artificially through pasture selection. Minimising disturbance can be facilitated temporary exclusion of native herbivores and control of domestic varieties, but should also include managing propagule dispersal down waterways and via other vectors. Additionally, the evidence that we found of gorse persisting not just in temperate environments but also in sub-humid and even tropical environments suggests that future managers of ‘open’ natural landscapes and agroecological zones in these climate regions should also be aware of the benefits of maintaining soil integrity and groundcover.

Ultimately, gorse is a symptom of a broader imbalance in the landscape, the same as all weeds of managed or modified environments. Though ecologically equipped to exploit these imbalances though its inherent life history characteristics, the ultimate solution to gorse management is to protect landscape function and go back to basics with respect to techniques. For gorse, the future is in what we already understand, but may not have put together, not in what we do not yet know, and could never hope to achieve.

## Figures and Tables

**Table 1:** Herbicide classes and application techniques evaluated in a global study of the success of management of common gorse.

Application	Class	Name	Efficacy (%)	Success ^1^	Source
Foliar	ACC‘ase inhibitors + synthetic auxins	Quizalofol, triclopyr, picloram, clopyralid, haloxyfop	0%–40% mortality	*	[58]
	Inhibitors of 5-enolpyruvyl shikimate-3 phosphate (EPSP) synthase	Glyphosate	55%–100% mortality	***	[9,56]
	Inhibitors of 5-enolpyruvyl shikimate-3 phosphate (EPSP) synthase + inhibiting cell division	Glyphosate + metsulfuronmethyl	55%–70% mortality	***	[9]
	Inhibitors of 5-enolpyruvyl shikimate-3 phosphate (EPSP) synthase + synthetic auxins	Glyphosate + picloram	88% mortality	****	[59]
	PS II inhibitors	Hexazinone terbuthylazine	29%–92% necrosis	***	[58,59]
	PS II inhibitors + synthetic auxins	Terbuthylazine + triclopyr + picloram	75% mortality	****	[59]
	Synthetic auxins	2,4-D, 2,4,5-T, dicamba, clopyralid, triclopyr, picloram	80%–100% mortality	****	[9,57,58,60]
	Other	Super-heated water	100% mortality	*****	[61]
Soil	Inhibits demethylation	Cyproconazole	53% seed viability	***	[17]
	PS II inhibitors	Bromoxynil	46% seed viability	**	[17]
	PSI inhibitors	Reglone, seed spray	0% seed viability	*****	[17]
	Synthetic auxins	MPCA, triclopyr, picloram, 2,4-D	32%–55% seed viability	**	[17]
Cut stump	ALS inhibitors	Imazapyr	100% mortality	*****	[9]
	Inhibitors of 5-enolpyruvyl shikimate-3 phosphate (EPSP) synthase	Glyphosate	65% mortality	***	[9]
	PSI inhibitors	Diquat	76% mortality	****	[20]
	PSI inhibitors + synthetic auxins	2,4,5-T + diquat	70% mortality	***	[20]
	Synthetic auxins	Picloram, triclopyr, 2,4-D, 2,4,5-T	89%–100% mortality	****	[9,61,62]

^1^ Success and efficacy: Completely effective (*****); effective, upper quartile (****); somewhat effective (50%–75%) (***); not very effective (25%–49%) (**); ineffective = bottom quartile (*).

## Appendix A

**Table A1:** Biological controls (phytophagous insects) and their success in reducing plant vigour and population size of common gorse.

Species	Location	Mortality or Loss of Vigour (%)	Success ^1^	Comments	Source
*Sericothrips staphylinus* (Haliday)	USA, Hawaii	100%	*****	Potted experiment kills mature plants in 2–3 years	[49]
	New Zealand	0%	*	Field study displaying no sign of impact on plant heath	[53]
		Low	*	Significantly reduced growth rates of small potted plants	[85]
	Australia	57%	***	Reduction in plant dry weight	[80]
		0%	*	No visible impact	[86]
		Low	*	Visible impact only at high population densities	[86]
*Pempelia genistella* (Duponchel)	USA, Hawaii	0%	*	Impact considered insignificant to the control of gorse, with no recorded impact	[55,87]
*Agonopterix ulicetella* (Stainton)	Chile	49%–88%	***	First year, 88%, second year, 49%, reduction of new shoots.	[49]
	Hawaii	Some	***	Recorded to cause extensive damage above 1000 m	[88]
	New Zealand	Some	***	Single larva able to kill five shoots; able to live in dense communities	[49]
	Australia	0%	*	Low population density, with no recorded impact on gorse	[54]
*Tetranychus lintearius* (Dulfour)	USA, Hawaii	37%–82%	***	37% reduced shoot elongation; 82% reduction in flowering	[5]
	New Zealand	Low	**	Decreased vigour and competitiveness through stress	[48]
		Some	**	Killed individual shoots; reduced growth rates	[42]
	USA, Oregon	1.5%–4.4%	*	Average relative mite damage ranged from 1.5% to 4.4%	[89]
		<50%	**	Average relative mite damage was ~7% with four plants suffering >50% damage, however, impact reduced by predatory mites	[50,89,90,91]
	Australia	36%–44%	**	Reduction in dry matter of 36%–44%, however, impact reduced due to predatory mites	[34,36]

^1^ Success and efficacy: Completely effective (*****); effective, upper quartile (****); somewhat effective (50%–75%) (***); not very effective (25%–49%) (**); ineffective = bottom quartile (*).

**Table A2:** Biological controls (seed-feeders) and their success in reducing plant vigour and population size of common gorse.

Species	Location	Efficacy	Success ^1^	Comments	Sources
*Cydia succedana* (Denis & Schiffermüller)	France	6%–47	*	Annual seed predation	[92]
	New Zealand	~45%	**	Best damaged recorded, predominantly in autumn and spring, coinciding with flowering)	[10]
		~62%	***	Reduction only recorded during spring flowering	[37]
		10%–20%	*	Destroys 10%–20% of the annual seed crop, only noticeable in spring, second moth generation in autumn is small, does little damage	[42]
		21%	*	3%–55% attack, with the majority of damage occurring in summer, with limited damage in spring or autumn	[38]
*Exapion ulicis* (Forster)	Chile	~98%	****	Weevils attack up to 98% of seed pods, reducing biomass, seed production, and seedling colonization	[52]
	France	31%–78%	***	Seed pod parasitisation	[40]
		14%–57%	**	Seed predation by weevils	[92]
	Great Britain	69.4%	***	69.4% of pods had most of the seeds destroyed, with site-specific variation of 0%–92%	[39]
	Hawaii	78%	****	Pods attacked	[88]
		59.4%	***	Increased 10%–20% per year reaching 59.4% predation after 9 years	[11]
		52%	***	Highly varied attack detection from 1.5%–80% average 52% fluctuating between sites and years	[93]
		84%	****	By 1984, the population had increased and 84% of the pods were infested compared to the initial 1.5% predation recorded in 1972	[94]
	New Zealand	>80%	****	Spring infestation of the pods in 1983/1994/1995	[42]
		90%	****	Recorded during peak spring flowering	[42]
		76%	***	Up to 76% spring/summer predation, yet highly variable	[10]
		54%	***	Spring seed predation	[37]
		6%–94%	***	Spring seed destruction: 6%~94% per plant	[43]
		36%	**	36% year-round infestation peaking at 64% in spring/early summer, reducing seed production in infected pods by 90%	[95]
	Australia	12%–55%	**	Annual reduction in seed production	[80]
*Exapion ulicis* and *Cydia succedana*	France	60%–80%	***	Year-round damage, in full sun across a range of plant densities	[96]
	New Zealand	~100%	*****	Spring-produced seed was attacked by both agents, with virtually all seed being destroyed Autumn-produced seed attacked by only Cydia, with about 10% of the seed being destroyed	[44]
		~90%	****	Reduced annual seed crop by up to 90%	[43]

^1^ Success and efficacy: Completely effective (*****); effective, upper quartile (****); somewhat effective (50%–75%) (***); not very effective (25%–49%) (**); ineffective = bottom quartile (*).

**Table A3:** Mycoherbicides and their success in reducing plant vigour and population size of common gorse.

Species	Location	Survival (%)	Success ^1^	Comments	Source
*Fusarium tumidu*	New Zealand	17%	****	100% seedling cover with *F. tumidum* caused mortality	[64]
		45%	***	Reduction in living shoots and stem	[64]
		15%	****	15% of pre-wounded young seedlings died from infection with older seedling recording a reduction in biomass (41%)	[35]
		55%–95%	**	All gorse seedlings were susceptible to the fungus, but younger plants were more easily killed, with survival increasing with plant age	[63]
		50%–83%	**	Significantly reduced emergence of gorse seedlings and further reduced both shoot and root dry weights by 42% and 56% respectively	[33]
*Chrondrostereum purpureum*	Canada	50%	***	Many cut stems re-sprouted, but were stunted, with the bio-herbicide killing approximately half the re-sprouts after two years	[8]
		40%	***	51% of cut stems re-sprouted, a 40% reduction from the control	[8]
	New Zealand	43%–44%	***	43%–44% reductions in regenerative shoot density	[64]
		28%–38%	***	Stump survival was reduced by 28%–38%	[64]

^1^ Success and efficacy: Completely effective (*****); effective, upper quartile (****); somewhat effective (50%–75%) (***); not very effective (25%–49%) (**); ineffective = bottom quartile (*).
